# Upregulation of the MYB2 Transcription Factor is Associated with Increased Accumulation of Anthocyanin in the Leaves of *Dendrobium bigibbum*

**DOI:** 10.3390/ijms21165653

**Published:** 2020-08-06

**Authors:** Gah-Hyun Lim, Se Won Kim, Jaihyunk Ryu, Si-Yong Kang, Jin-Baek Kim, Sang Hoon Kim

**Affiliations:** 1 Advanced Radiation Technology Institute, Korea Atomic Energy Research Institute (KAERI), Jeongeup 56212, Korea; kah7702@kaeri.re.kr (G.-H.L.); sewonk@korea.kr (S.W.K.); jhryu@kaeri.re.kr (J.R.); sykang@kaeri.re.kr (S.-Y.K.); jbkim74@kaeri.re.kr (J.-B.K.); 2 National Institute of Agricultural Sciences, Rural Development Administration, Jeonju 54874, Korea

**Keywords:** anthocyanin, MYB2, orchid, *Dendrobium bigibbum*, γ-irradiation

## Abstract

Orchids with colorful leaves and flowers have significant ornamental value. Here, we used γ-irradiation-based mutagenesis to produce a *Dendrobium bigibbum* mutant that developed purple instead of the normal green leaves. RNA sequencing of the mutant plant identified 2513 differentially expressed genes, including 1870 up- and 706 downregulated genes. The purple leaf color of mutant leaves was associated with increased expression of genes that encoded key biosynthetic enzymes in the anthocyanin biosynthetic pathway. In addition, the mutant leaves also showed increased expression of several families of transcription factors including the *MYB2* gene. Transient overexpression of *D. biggibum* *MYB2* in *Nicotiana benthamiana* was associated with increased expression of endogenous anthocyanin biosynthesis genes. Interestingly, transient overexpression of orthologous *MYB2* genes from other orchids did not upregulate expression of endogenous anthocyanin biosynthesis genes. Together, these results suggest that the purple coloration of *D. biggibum* leaves is at least associated with increased expression of the *MYB2* gene, and the *MYB2* orthologs from orchids likely function differently, regardless of their high level of similarity.

## 1. Introduction

In Orchidaceae, *Dendrobium* species are one of the most popular orchids known for their medicinal and commercial value in potted and cut flower industries [1]. The *Dendrobium* genus contains approximately 1800 species and are mainly distributed throughout Asia and the South Pacific [2]. *Dendrobium catenatum* (also named *Dendrobium officinale*), *Dendrobium nobile*, and *Dendrobium candidum* are used in herbal medicines in many Asian countries [3]. Moreover, the *Dendrobium* genus is known for its valuable floral traits including colors, morphologies, and scent and *Dendrobium* species are regarded as some of the important commercial cut flowers. A variety of *Dendrobium* hybrids have been created that have improved flower colors. However, limitation of genetic resources in *Dendrobium* limits the extent to which flower color can be modified. 

*D. bigibbum* is an epiphytic or lithophytic orchid that contains cylindrical pseudobulbs, each having between three and five green or purplish leaves and arching flowering stems with up to 20 usually lilac-purple flowers. The *D. bigibbum* plants containing purple spots on their leaves are very popular in the commercial market. Although colorful leaves and flowers add significant ornamental values to orchids, our understanding of the differential pigmentation in *D. bigibbum* remains limited. Natural agents extracted from various parts of *Dendrobium* contain bioactive substances, such as phenolic compounds, anthocyanins, and polysaccharides [4,5,6]. Many of these phenolic compounds and anthocyanins have well-known antioxidant activities [7] and contribute to leaf and flower coloration [8]. Anthocyanins are water-soluble, which are present in the vacuoles of plant epidermal cells and impart an orange, red, or blue color to flowers, fruits, stems, leaves, and roots [9]. Anthocyanin biosynthesis is a well-studied secondary metabolic pathway in plants that involves the conversion of phenylalanine into 4-coumaryl-CoA, followed by their conversions to flavonoid compounds. Studies in antirrhinum [10], petunia [11,12], maize [13,14], *Brassica* [15,16], and *Arabidopsis* [17,18] have identified genes that regulate anthocyanin production, and these can be broadly classified into two major groups. The first group consists of enzymes that participate in anthocyanin biosynthesis, including phenylalanine ammonia-lyase (PAL), chalcone synthetase (CHS), chalcone isomerase (CHI), flavanone 3-β-hydroxylase (F3H), dihydroflavonol 4-reductase (DFR), anthocyanin synthase (ANS), and UDP-glucose flavonoid 3-O-glucosyl transferase [19]. Loss-of-function mutations in *CHS*, *CHI*, *F3H*, *DFR*, or *ANS* abolish anthocyanin biosynthesis, and plants harboring these mutations often produce colorless tissues [20,21,22,23,24]. Anthocyanin accumulations in green and red leaves of *Dendrobium officinale* stems have been associated with *ANS* and UDP-glucose flavonoid 3-*O*-glucosyl transferase expression [25]. Moreover, Yu et al. suggested that among anthocyanins, delphinidin 3,5-*O*-diglucoside and cyanidin 3-*O*-glucoside may be responsible for the red peel color of *D. officinale* [25]. The second group contains MYBs, basic helix-loop-helixes (bHLHs), or WD40 repeat transcription factors (TFs) that regulate the expression levels of genes involved in anthocyanin biosynthesis [26,27]. Earlier studies on *Arabidopsis* and *Medicago truncatula* indicated that MYB2 acts as a transcriptional repressor of anthocyanin biosynthesis and that the overexpression of MYB2 abolishes anthocyanin biosynthesis [28,29]. However, the overexpression of orchid *MYB2* in petunia results in increased petal pigmentation [11]. Likewise, the transient overexpression of *Phalaenopsis equestris MYB2* positively regulates anthocyanin pigmentation and is associated with the increased expression of downstream genes *PeF3H5*, *PeDFR1*, and *PeANS3* [30]. Conversely, silencing of *PeMYB2* results in reduced anthocyanin accumulation [31]. Thus, depending on the plant system, MYB2 appears to serve as either a negative or positive regulator of anthocyanin biosynthesis.

In this study, we characterized an orchid mutant that was isolated on the basis of its unusual leaf color. The *D. bigibbum* mutant accumulated higher levels of anthocyanin, which in turn was associated with the increased expression of genes regulating anthocyanin biosynthesis. This also included the *MYB2* gene, which, when transiently expressed in a heterologous system, led to induction of genes associated with anthocyanin biosynthesis. 

## 2. Results

### 2.1. The Purple Mutant of D. Bigibbum Accumulates Higher Levels of Anthocyanin

**Figure 1:**
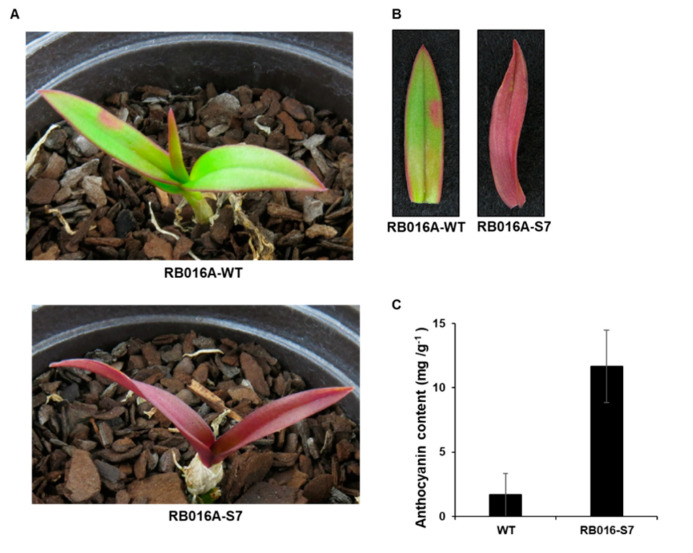
Images of *Dendrobium bigibbum* and the anthocyanin contents in the leaves. (**A**) Morphological phenotypes of typical wild type (WT) and RB016-S7 mutant *D. bigibbum* plants. (**B**) Relative anthocyanin contents in the WT and RB016-S7 mutant. Error bars represent standard deviations (*n* = 3). The experiment was repeated three times with similar results. (**C**) The number of up- and downregulated differentially expressed genes (DEGs) in the WT 3 versus RB016-S7 mutant comparison.

We used γ-irradiated *D. bigibbum* rhizomes to produce a mutant that developed purple leaves in comparison to the green leaves seen on wild type (WT) plants (Figure 1A,B) (Figure S1). This mutant, designated as RB016-S7, was propagated through four generations of tissue culturing. To determine whether the purple coloration of the mutant’s leaves was associated with anthocyanin pigmentation, we used a pH-differential-based method to quantify the anthocyanin content. The anthocyanin content in the purple leaves (11.68 mg/g dry weight) was ~7.0-fold higher than in the green leaves (1.66 mg/g dry weight) (Figure 1C). Thus, the purple coloration of RB016-S7 leaves was likely associated with the increased biosynthesis of anthocyanins.

**Figure 2:**
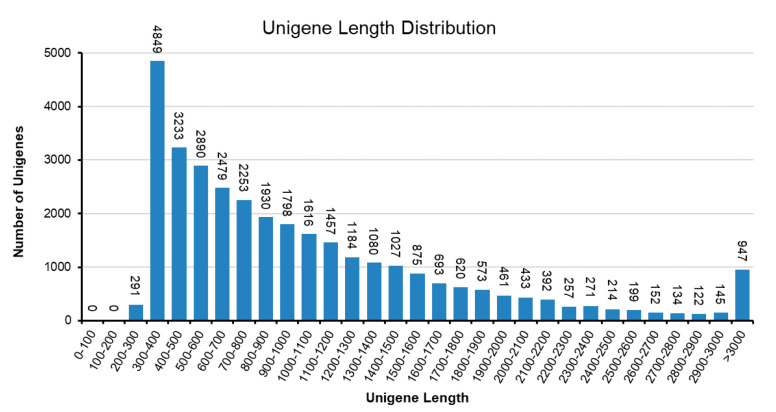
Sequence length distribution of the unigenes in *D. bigibbum* transcriptomes. The *x*-axis indicates unigene length intervals from 200 bp to >3000 bp. The *y*-axis indicates the number of unigenes of each given length.

**Figure 3:**
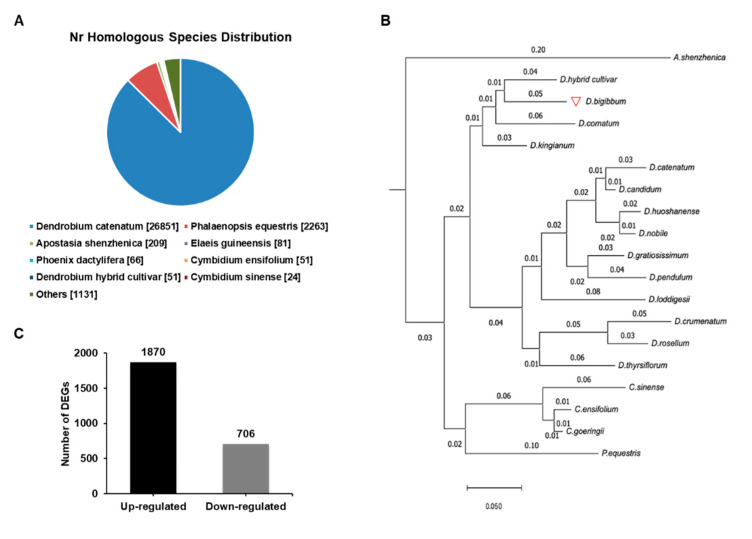
Species distribution of the BLAST search results in the nr database. (**A**) A cut off E-value of 10^−5^ was used. Different species are indicated by different colors. (**B**) A reference phylogenetic tree derived from rDNA ITS 2 sequences of 14 species of *Dendrobium*, 3 species of *Cymbidium*, *Apostasia shenzhenica*, and *Phalaenopsis equestris*. (**C**) The number of up- and downregulated differentially expressed genes (DEGs) in the wild type versus RB016-S7 mutant comparison.

To understand the biochemical basis of the increased anthocyanin production in the RB16-S7 mutant, we analyzed genome-wide changes in gene expression. Total RNAs from WT and RB016-S7 leaves were used to construct six cDNA libraries that were sequenced using the Illumina HiSeq 2500 platform. After filtering and quality trimming the raw reads, we obtained 47–66 million high quality reads. Using Trinity, the clean reads from the six libraries were assembled into 110,104 transcripts, with an average length of 1116 bp, and these were then assembled into 32,575 unigenes, with an average length of 1048 bp (Table 1). The sequence length distribution of unigenes showed that 8373 unigenes (25.7%) ranged from 100 to 500 bp, 11,350 unigenes (34.8%) ranged from 501 to 1000 bp, and 6488 unigenes (19.91%) had lengths of more than 1500 bp (Figure 2). The 30,714 unigenes were matched with the non-redundant (nr) database, and among these, 26,851 unigenes matched sequences from *Dendrobium catenatum*, followed by *Phalaenopsis equestris* (2263) and *Apostasia shenzhenica* (209) (Figure 3A). Furthermore, this was consistent with the phylogenetic analysis carried out among native *Dendrobium spp*, *Cymbidium spp*, *P. equestris* and *A. shenzhenica* orchids, which, as expected, showed relatedness among *Dendrobium spp* (Figure 3B). 

### 2.2. Functional Annotation and Classification

In the Gene Ontology (GO) analysis, 17,498 unigenes (53.71%) were assigned to three GO terms and were categorized into 41 functional groups (FDR < 0.05) (Table S1). The GO assignments were divided into three categories: biological process (BP), cellular component (CC), and molecular function (MF). Among these, 10,569 unigenes (32.4%), 9195 unigenes (28.2%), and 12,401 unigenes (38%), were assigned to BP, CC, and MF, respectively. In the BP category, the predicted proteins were mainly distributed in metabolic process (30.61%) and cellular process (28.36%), followed by biological regulation (7.34%), localization (6.87%), and regulation of biological process (6.09%). Predicted proteins assigned to the CC category were mainly associated with cellular anatomical entity (55.61%), intracellular (30.19%), and protein-containing complex (12.84%). Furthermore, in the MF category, the most heavily represented groups were linked to catalytic activity (47.13%), binding (40.78%), and transporter activity (5.04%) (Figure S2). 

To predict and classify the gene functions, we queried all the unigenes against the evolutionary genealogy of genes: Non-supervised Orthologous Groups (eggNOG) (v4.5) database. This database contains the functional descriptions and classifications of the orthologous proteins, including Clusters of Orthologous Groups and euKaryotic Orthologous Groups. This analysis allowed us to allocate 27,963 unigenes to 25 eggNOG classifications. Among them, the eggNOG category of functional unknown (S, 27.88%) represented the largest group, followed by signal transduction mechanisms (T, 8.58%), posttranslational modification, protein turnover, chaperones (O, 8.13%), transcription (K, 8.09%), and carbohydrate transport and metabolism (G, 5.70%) (Figure S3). 

Next, we mapped the assembled unigenes to the reference anthocyanin pathways, including metabolism, genetic information processing, environmental information processing, and cellular processes, in the KEGG (http://www.kegg.jp/kegg/pathway.html). The 6314 unigenes were assigned to 394 KEGG sub-pathways (Table S2). These pathways included KEGG orthology (KO) entries for metabolism (3503 KOs), genetic information processing (950 KOs), environmental information processing (488 KOs), cellular processes (702 KOs), and organismal systems (671 KOs) (Figure S4). 

### 2.3. Analysis of Differentially Expressed Genes (DEGs) Associated with Anthocyanin Biosynthesis 

A total of 2513 DEGs (FDR < 0.05) were identified between the WT and RB016-S7 mutant. Compared with WT, 1870 and 706 genes were up- and downregulated in the RB016-S7 mutant, respectively (Figure 3C; Table S3). The top 20 significant pathways for the up- and downregulated genes were selected for further analysis. The upregulated genes were mainly enriched in ribosome biogenesis, MAPK signaling pathway, plant–pathogen interaction, plant hormone signal transduction, phenylpropanoid biosynthesis, starch and sucrose metabolism, and flavonoid biosynthesis (Table 2). The 20 significant pathways for the downregulated genes are listed in Table 3. The downregulated genes were mainly enriched in folate biosynthesis, starch and sucrose metabolism, plant hormone signal transduction, and phenylpropanoid biosynthesis.

### 2.4. Analysis of Anthocyanin Biosynthetic Genes in Identified DEGs

**Figure 4:**
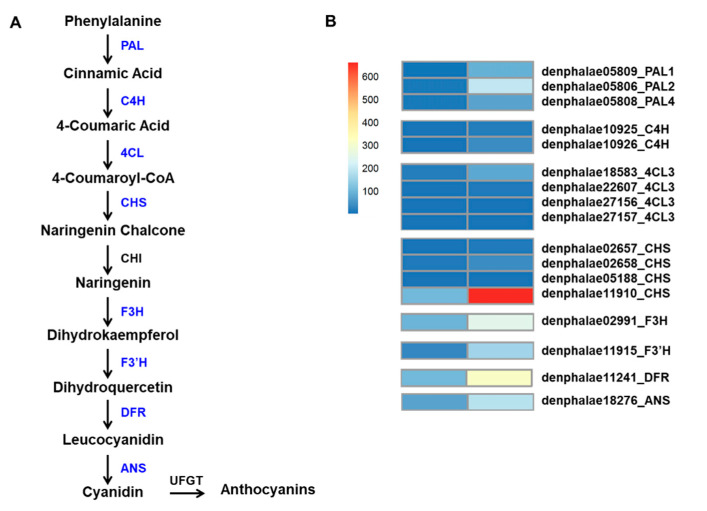
Flavonoid–anthocyanin biosynthetic genes in *D. bigibbum*. (**A**) The differentially expressed genes (DEGs) between the WT and RB016-S7 mutant found in leaves are highlighted in blue. Phenylalanine ammonia-lyase, PAL; cinnamic acid 4-hydroxylase, C4H; 4-coumarate CoA-ligase, 4CL; chalcone synthase, CHS; chalcone isomerase, CHI; flavanone 3-hydroxylase, F3H; flavonoid 3′-monooxygenase, F3′H; dihydroflavonol 4-reductase, DFR; and anthocyanidin synthase, ANS. (**B**) Expression profiles determined using fragments per kilobase of transcript per million mapped reads (FPKM) values obtained from RNA-Seq data. Expression values (as FRKM) were not scaled per row to allow the visualization of original FPKM values among samples. The heatmap was generated using the R package pheatmap.

The mutant showed an increased accumulation of anthocyanin; therefore, we used a KEGG functional enrichment to search for genes associated with anthocyanin biosynthesis among the 2513 DEGs. A total of 17 DEGs, encoding eight key enzymes, were identified, and they were three *PAL* genes (*PAL1*: denphalae05809, *PAL2*: denphalae05806, and *PAL4*: denphalae05808), two cinnamic acid 4-hydroxylase genes (*C4H*: denphalae10925 and denphalae10926), four 4-coumarate CoA-ligase genes (*4CL*: denphalae18583, denphalae22607, denphalae27156, and denphalae27157), four *CHS* genes (denphalae02657, denphalae02658, denphalae05188, and denphalae11910), and one gene each of *F3H* (denphalae02991), flavonoid 3′-monooxygenase (*F3′H*: denphalae11915), *DFR* (denphalae11241), and *ANS* (denphalae18276). All these DEGs were significantly upregulated in the RB016-S7 mutant compared with WT (Table 4). Among other notable genes that were upregulated in RB016-S7 were TFs that belonged to WRKY (33 genes), MYB (20 genes), bHLH (23 genes), and WD40 (1 gene) groups. Among these, *DbMYB2*, *-4*, *-30*, and *-44*, as well as *DbbHLH1*, *-62*, *-96*, *-114*, and *-148*, were highly expressed in the RB016-S7 mutant (Table S4). The expression patterns of the anthocyanin biosynthetic genes were consistent with the increased anthocyanin levels in the RB16-S7 mutant (Figure 4A,B).

### 2.5. Quantitative Real-Time PCR (qRT-PCR) Analysis of the Genes Involved in Anthocyanin Biosynthesis

**Figure 5:**
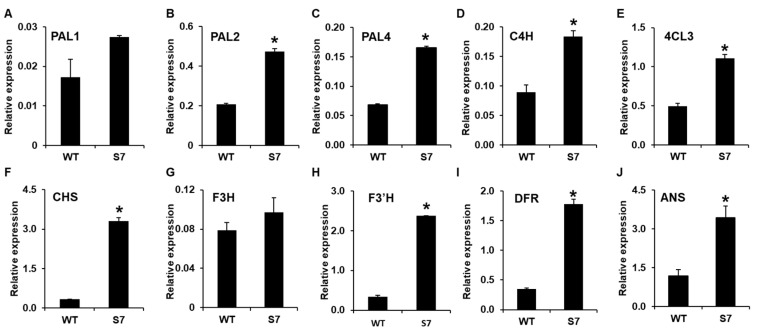
qRT-PCR analysis of 10 genes showing altered expression levels in the RNA sequencing (RNA-Seq) analysis. The genes were associated with anthocyanin biosynthesis. More specifically, (**A**–**J**) indicate the relative expression levels of *PAL1*, *PAL2*, *PAL3*, *C4H*, *4CL*, *CHS*, *F3H*, *F3′H*, *DFR*, and *ANS*, respectively. The elongation factor 1-alpha (*EF1a*) gene served as an internal control. Error bars indicate standard deviations (*n* = 3). The experiment was repeated three times with similar results. Asterisks denote a significant difference between respective WT and RB016-S7 mutant leaves samples (*t*-text, *p* < 0.0001).

To confirm the RNA-Seq data, we first selected 10 candidate genes associated with anthocyanin biosynthesis and analyzed their expression levels using qRT-PCR. The qRT-PCR analysis confirmed ~1.5-, 2.3-, and 2.4-fold higher levels for *PAL1*, *PAL2*, and *PAL4*, respectively, in the RB016-S7 mutant compared with the WT. In addition, the qRT-PCR analysis showed that *C4H*, *4CL*, and *CHS* were induced ~2-, 3.3-, and 10-fold in the RB016-S7 mutant compared with the WT. Similarly, *F3’H*, *F3H*, *DFR*, and *ANS* were induced ~1.2-, 7.2-, 5.0-, and 3-fold in the RB016-S7 mutant compared with the WT (Figure 5). The qRT-PCR data were consistent with results obtained from the RNA-Seq data. Thus, the purple pigmentation in RB016-S7 may be associated with the increased expression levels of genes involved in anthocyanin biosynthesis. 

Next, we analyzed the expression levels of regulatory genes associated with anthocyanin biosynthesis. The RNA-Seq dataset showed that *DbMYB2*, *-30*, and *-44* were highly upregulated in the RB016-S7 mutant compared with the WT, while the expression of *DbMYB75* was not significantly different from in WT plants. Notably, the qRT-PCR analysis was only able to confirm a ~13-fold induction in *DbMYB2*, while the expression of *DbMYB30*, *-44*, and *-75* remained at WT levels.

**Figure 6:**
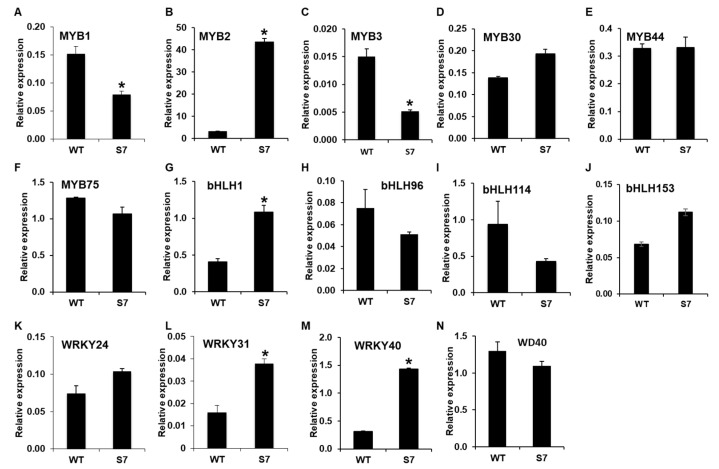
qRT-PCR analysis of 14 genes showing altered expression levels in the RNA-Seq analysis. The relative expression levels of transcription factor genes in the leaves. More specifically, (**A**–**N**) indicate the relative expression levels of *MYB1*, *MYB2*, *MYB3*, *MYB30*, *MYB44*, *MYB75*, *bHLH1*, *bHLH96*, *bHLH114*, *bHLH153*, *WRKY24*, *WRKY31*, *WRKY40*, and *WD40*. The *EF1a* gene served as an internal control. Error bars indicate standard deviations (*n* = 3). The error bars indicate SD (*n* = 3). Results are representative of two independent experiments. Asterisks denote a significant difference between respective WT and RB016-S7 mutant leaves samples (*t*-test, *p* < 0.0001).

Comparisons of expression levels of genes encoding bHLH TFs showed that only *DbbHLH1* was expressed at higher levels in RB016-S7 than WT plants. In comparison, *DbbHLH96*, *-114*, and *-153* showed WT-like expression levels. *DbWD40*, which showed a 67.97% identity to the *Arabidopsis* ortholog *AtTTG1*, had a WT-like expression level [32]. A recent report also suggests roles for WRKY TFs in anthocyanin biosynthesis. RNA-Seq data showed that several WRKY TFs were highly expressed in the RB016-S17 mutant compared with WT. However, the qRT-PCR analysis was only able to confirm ~1.5–3-fold inductions of *DbWRKY24*, *WRKY31*, and *WRKY40* genes (Figure 6). Thus, only a select group of TFs were upregulated in the mutant plant, and these, in turn, could play roles in the regulation of genes involved in anthocyanin biosynthesis. 

### 2.6. DbMYB2 Positively Regulates Anthocyanin Biosynthesis

**Figure 7:**
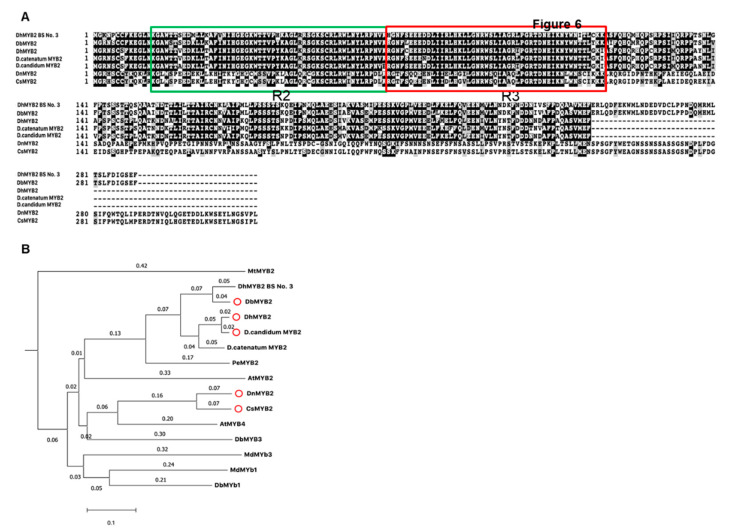
Sequence alignments and phylogenetic tree of DbMYB2 from orchids with known R2R3-MYBs domain. (**A**) Alignments of the full-length deduced amino acid sequences of DbMYB2 with other R2R3-MYBs present in other orchids. (**B**) Phylogenetic relationship of DbMYB2 with known anthocyanin MYB regulators from other orchid species.

Increased expression of *DbMYB2* in the RB016-S17 mutant suggested that MYB2 could positively regulate expression of anthocyanin genes and thereby anthocyanin levels. This is further supported by an earlier study that showed that *Dendrobium* hybrid MYB2 positively regulated anthocyanin pigmentation in *Dendrobium* petals. Amino acid alignment of DbMYB2 with DhMYB2 BS No.3 [33] showed ~92% identity. Likewise, amino acid alignment of MYB2 orthologs from *D.* hybrid, *D. candidum*, *D. nobile*, and *Cymbidium sinense* showed ~80%, ~80%, ~62%, and 63% identity, respectively, with DbMYB2 (Figure 7A). The amino acid alignment showed that the R2R3 repeat region was highly conserved among various MYB proteins *(*Figure 7A). Phylogenetic analysis between these MYB proteins placed DbMYB2, DhMYB2, and DcMYB2 in the same clade (Figure 7B).

**Figure 8:**
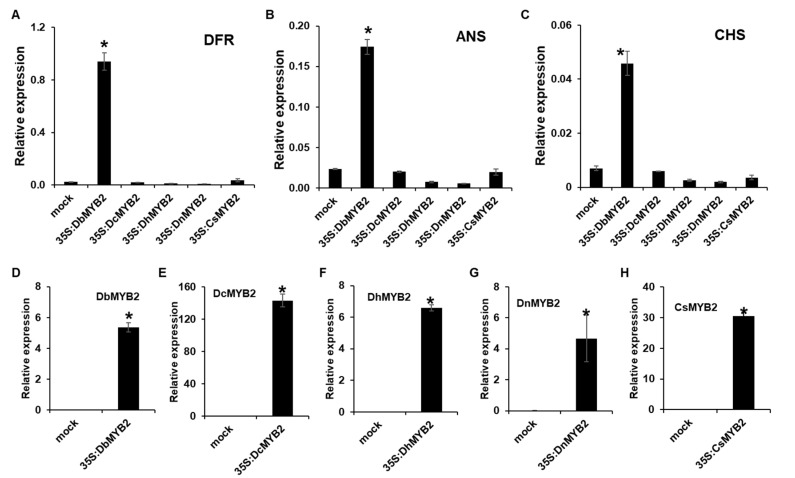
Transient expression of DbMYB2s were associated with increased expression of ANS, DFR, and CHS in *N. benthamiana*. (**A**–**C**) Real-time quantitative RT-PCR showing relative expression levels of *NbDFR*, *NbANS*, and *NbCHS* genes in *N. benthamiana* transiently overexpressing *MYB2* genes from different orchids. (**D**–**H**) Real-time quantitative RT-PCR showing relative expression levels of *MYB2* genes in *N. benthamiana* plants transiently overexpressing *MYB2* orthologs from different orchids. The error bars indicate SD (*n* = 4). Results are representative of three independent experiments. Asterisks denote a significant difference with mock and MYB2 overexpressed plants (*t*-test, *p* < 0.0001).

To determine whether increased expression of DbMYB2 positively regulated expression of anthocyanin genes, we expressed *MYB2* genes from *D. bigibbum*, *D. candidum*, *D. nobile*, *D.* hybrid, and *C. sinense* in *Nicotiana benthamiana* and evaluated expression of *N. benthaminana* genes *ANS*, *DFR*, and *CHS*, which are associated with anthocyanin biosynthesis. All the *MYB2* genes showed varying levels of increased expression at 36 h post-agroinfiltration (Figure 8D–H). Interestingly, however, only transient expression of *DbMYB2* was associated with increased expression of *ANS*, *DFR*, and *CHS* in *N. benthamiana* (Figure 8A–C). These results strongly suggest that DbMYB2 positively regulates expression of genes associated with anthocyanin biosynthesis, and that higher anthocyanin levels in the RB016-S17 mutant are likely due to higher expression levels of *DbMYB2*. Inability of other *MYB2* orthologs to increase expression of *ANS*, *DFR*, and *CHS* suggests that, regardless of their homology, the *MYB2* orthologs function differently.

## 3. Discussion

Anthocyanins are pigments that confer color to various plant parts [34]. The color is determined by the composition and concentration of pigments, which vary greatly among plant species [35]. Cyanidin-3-glucoside is a major anthocyanin found in most plants [36]. Other common anthocyanin pigments present in plants include delphinidin, pelargonidin, peonidin, malvidin, and petunidin. Earlier studies on *Dendrobium* orchids primarily focused on anthocyanin profiles in flowers and stems, which contain pelargonidin, cyanidin, peonidin, delphinidin, and/or malvidin [24]. In contrast, we were only able to detect malvidin in the leaves of *D. bigibbum* (data not shown), and its levels were associated with increased purple pigmentation in the RB016-S7 mutant’s leaves. Thus, anthocyanin pigments present in leaves versus flowers and stems might be associated with the specific genes expressed in these tissues. We determined that the increased anthocyanin accumulation in RB016-S7 was associated with increased expression levels of *PAL*, *CHS*, *F3′H*, and *DFR* genes that are involved in anthocyanin biosynthesis. Although the anthocyanin biosynthetic genes are well-conserved, the timing, level, and spatial distribution of anthocyanin biosynthesis are primarily determined by TFs. 

A recent study offered useful insights into the functions of WRKY TFs in anthocyanin biosynthesis [37], which in turn regulates the MYB/bHLH/WD40complex [27,38,39,40]. Our analysis also identified 33 WRKY-, 20 MYB-, 23 bHLH- and 1WD40-encoding genes that were differentially expressed in the RB016-S7 mutant leaves. It is possible that these TFs regulate the expression of one or more genes involved in the anthocyanin pathway (Table S5). An example of complex regulation underlying anthocyanin biosynthesis includes the feed-forward loop mechanism in which TFs regulate each other and jointly regulate target genes [28]. In *Dendrobium* hybrid petals, *DhMYB2* and *DhbHLH1* TFs play regulatory roles in the anthocyanin biosynthetic pathway [33]. Consistent with this finding, we determined that the expression levels of *DbMYB2* and *DbHLH1* were significantly higher in the RB016-S7 mutant. Notably, the *A. thaliana* and *M. truncatula* MYB2 proteins act as transcriptional repressors of anthocyanin biosynthesis, and the overexpression of either MYB2 abolishes anthocyanin biosynthesis [28,29]. Likewise, heterologous overexpression of *Malus domestica MYB3* (*MdMYB3*) in *Nicotiana tabacum* is associated with increased anthocyanin biosynthesis [41]. Thus, orthologs of MYB and possibly other genes involved in anthocyanin biosynthesis may play opposite roles in different plants. This was further evident in our analysis, which showed that increased expression of *MYB2* in RB016-S7 plants positively correlated with anthocyanin biosynthesis. This was further consistent with our result that heterologous overexpression of *D. bigibbum MYB2* in *N. benthamiana* led to increased expression of *ANS*, *DFR*, and *CHS* genes. Interestingly, transient overexpression of *MYB2* orthologs from other *Dendrobium spp.* or *C. sinense* did not alter expression of *ANS*, *DFR*, and *CHS* genes. These results strongly suggest that DbMYB2 positively regulates expression of genes associated with anthocyanin biosynthesis, even though DbMYB2 showed high levels of homology with other MYB2 orthologs. Thus, subtle changes in MYB2 sequence are likely sufficient to alter their function. It is possible that MYB2 in other orchids could regulate anthocyanin biosynthesis genes by serving as a part of the bigger complex that contains other factors like bHLHs or WD40. Deciphering the exact biochemical functions of various TFs involved in anthocyanin biosynthesis in *D. bigibbum* will require more detailed analyses of these proteins. 

## 4. Materials and Methods

### 4.1. Plant Materials

Protocorm-like bodies (PLBs) of *Dendrobium bigibbum* var. compactum were cultured on pH5.3 Hyponex medium (6.5:6.0:19:0 N:P:K; Hyponex Japan Corp., Ltd, Osaka, Japan) supplemented with sucrose (3% *w/v*) and agar (0.4% *w/v*) (Duchefa Biochemie B.V., Haarlem, The Netherlands). PLBs were cultured in 220 ml glass jars containing 30ml medium, which were closed with semipermeable plastic caps. All the cultures were maintained at 22–25 °C and >60% humidity. Plants were grown under white fluorescent light (PPFD = 50 μmol/m^2^/s) with a 16-h illumination and 8-h dark photoperiod.

### 4.2. γ-Irradiation of *in Vitro* Shoot Cultures

Six-month-old in vitro regenerated shoots of approximately 3 cm in length were exposed to γ-radiation using 60Co γ-irradiator (60 Gy/24 h) at the Korea Atomic Energy Research Institute, Jeongeup, Korea [42]. The first vegetative generation in which treatment was performed was referred to as M1V1. The study continued until the fourth generation (M1V4) to confirm the stability of the induced traits. The purple-colored leaf mutant RB016-S7 was obtained and its physiological traits were analyzed.

### 4.3. RNA Extraction and Quantitative Real-Time PCR (qRT-PCR) Analysis

Total RNA was extracted from the WT and the RB016-S7 plants using an RNeasy plant mini kit (Qiagen, Hilden, Germany), following the manufacturer’s instructions. The RNA concentration and quality of each sample were determined using a Nanodrop 2000 spectrophotometer (Thermo Fisher Scientific, Waltham, MA, USA) and agarose electrophoresis, respectively. The cDNA was transcribed from 500 ng of total RNA using a ReverTra Ace-α-kit (Toyobo Co. Ltd, Osaka, Japan). The qRT-PCR was performed with a CFX96 touch real-time PCR detection system (Bio-Rad, Hercules, CA, USA) using iQ™ SYBR^®^ Green supermix (Bio-Rad, Hercules, CA, USA). The *D. bigibbum* actin gene was used as an internal control, and the 2^−ΔΔCt^ method was used to analyze differential expression levels. Cycle threshold values were calculated using CFX Manager 3.1 software (Bio-Rad, Hercules, CA, USA). Gene-specific primers are listed in Table S5.

### 4.4. Measurement of Total Anthocyanin Content

After harvesting the leaves, the samples were freeze-dried and subjected to solvent extraction using a solution of 85% ethanol acidified with 15% 1.5 N HCl. The samples were incubated at 4 °C for 24 h. Samples were diluted in two buffer solutions: potassium chloride buffer 0.025 M (pH 1.0) and sodium acetate buffer 0.4 M (pH 4.5). Absorbance was measured via spectrophotometer at 510 and 700 nm after 15 min of incubation at room temperature, respectively. Absorbance was calculated as

(1) $$\begin{array}{c} \mathrm{Anthocyanin}\;\mathrm{pigment}\;\left(\mathrm{cyanidin}-3-\mathrm{glucoside}\;\mathrm{equivalents},\mathrm{mg}/L\right)\\ =\frac{A\times \mathrm{MW}\times \mathrm{DF}\times 10^{3}}{\varepsilon \times 1} \end{array}$$

A = (*A*510 nm − *A*700 nm) pH1.0 − (*A*510 nm − *A*700 nm) pH4.5; MW (molecular weight) = 449.2 g/mol for cyanidin-3-glucoside (cyd-3-glu); DF = dilution factor; l = pathlength in cm; ε = 26,900 molar extinction coefficient, in L·mol^−1^·cm^−1^, for cyd-3-glu; and 10^3^ = factor for conversion from g to mg [43].

### 4.5. RNA-Seq Analysis, De Novo Assembly, and Unigene Generation

The cDNA libraries were prepared independently from both WT and RB016-S7 leaves. Low quality and duplicated reads, as well as adapter sequences, were removed from RNA-seq raw data using Trimmomatic with default parameters [44]. The de novo assembly was performed using Trinity (ver. 2.8.4) with default parameters [45]. Afterwards, redundant sequences were removed from the assembled transcript sequences using cd-hit-est (ver. 4.7) with a similarity threshold of 90% (i.e., removing similar sequences sharing more than a 90% identity), generating nr transcript sequences. Protein coding sequences (CDSs) were predicted and extracted from the nr transcript sequences using TransDecoder (ver. 5.5) with a parameter of selection of the longest CDS by comparison with Pfam database. The collection of extracted CDSs was designated as the unigene set and used for further analyses. The completeness of the unigene set was validated by analysis with Benchmarking Universal Single-Copy Orthologs (ver. 3.1.0) [46].

### 4.6. Functional Annotation of Unigenes

Sequences homologous to unigenes were identified using BLASTP analyses (cutoff e-value 1e-5) against the NCBI nr protein database. The GO terms, and eggnog (ver. 3.0) and KEGG pathways, were assigned to the unigenes based on BLASTP results using the Blast2GO program (ver. 5.2.5). Conserved domains in the unigene sequences were identified using InterProScan program (ver. 5.34-73.0) with default parameters. In addition, a KEGG pathway analysis was also performed with the KEGG Automatic Annotation KAAS Server using the single-directional best hit method and searching against representative gene sets from both eukaryotes and monocots. 

### 4.7. Expression Profiling of Unigenes

Trimmed high-quality RNA-seq reads were mapped on the unigene sequences using BWA (ver. 0.7.17-r1188) [47] and then, RNA reads mapped on unigene sequences were counted using SAMtools (ver. 1.9) [48]. Fragments per kilobase of transcript per million mapped reads values were calculated using the number of RNA-seq reads mapped on unigene sequences and used for the expression profiling of unigenes.

### 4.8. Identification of DEGs between the WT and RB016-S7 Mutant

The bioconductor package DESeq (ver. 1.22.1) was used to identify DEGs between samples [49]. Genes showing over two-fold expression changes with *p*-values of less than 0.05 were considered DEGs. The GO enrichment analysis was performed for the DEGs using Fisher’s exact test with an adjusted *p*-value of 0.05 in the Blast2GO program (ver. 5.2.5).

### 4.9. Analysis of Unigenes Involved in Anthocyanin Biosynthesis

For anthocyanin biosynthesis, unigenes assigned to the anthocyanin biosynthetic reference pathway from the KEGG pathway analysis were first selected. In addition, BLASTP searches (cutoff e-value: 1e-10) were performed using 40 *Arabidopsis* genes involved in anthocyanin biosynthesis [50] as queries, and then, unigenes with high similarity levels (≥ 60% identity and ≥ 80% alignment length) to the query sequences were selected as candidate unigenes that could be involved in anthocyanin biosynthesis. Expression values (fragments per kilobase of transcript per million mapped reads) for the genes were retrieved from expression profiles of the unigenes set and used for generating heatmaps using the R -package pheatmap (ver. 1.0.12).

### 4.10. Cloning of Orchid MYB Genes

The full-length *MYB2* cDNA (denphalae23719) was PCR-amplified from *D. bigibbum* leaves and cloned into the Gateway binary vector pMDC32 vector, under the 35S CaMV promoter. The primers used for amplification of *MYB2* sequences are listed in Table S5. All the amplified products were sequenced.

### 4.11. Agroinfiltration of N. Benthamiana

The pMDC32-MYB2 plasmids were transformed into *A. tumefaciens* strains LBA4404 via the freeze–thaw method [51]. Agrobacteria were grown in the LB medium supplemented with 100 mg/L kanamycin and incubated at 28 °C with shaking. Bacteria were pelleted by centrifugation (14,000*g* for 5 min) and resuspended to an OD_600_ = 0.8 in a buffer containing 10 mM MES pH 5.6, 10 mM MgCl_2_, and 200 μM acetosyringone. Cultures were then incubated for 2–4 h at room temperature. Bacteria were infiltrated into the underside of *N. benthamiana* leaves using a needleless 1 ml syringe. The agroinfiltrated plants were kept in the growth chamber maintained at 23 °C with a 16-h photoperiod.

## Figures and Tables

**Table 1:** Summary of RNA sequencing and de novo transcriptome assembly results.

Sequences	Control 1	Control 2	Control 3	RB016-S7-1	RB016-S7-2	RB016-S7-3
**BEFORE TRIMMING**						
Total nucleotides (bp)	4,962,911,568	5,845,449,712	5,115,158,264	4,956,765,904	5,110,844,048	4,343,315,032
Number of raw reads	65,301,468	76,913,812	67,304,714	65,220,604	67,247,948	57,148,882
**AFTER TRIMMING**						
Total nucleotides (bp)	4,288,037,900	4,994,465,471	4,370,988,523	4,215,819,862	4,288,137,551	3,561,476,236
Number of clean reads	56,856,166	66,205,490	57,973,956	55,934,410	56,856,134	47,287,332
GC content (%)	46.42	44.22	45.73	47.06	46.79	46.55
Q30 percentage (%)	95.67	95.86	95.56	95.43	95.68	95.22
**AFTER ASSEMBLY**						
Number of transcripts in the combined data		110,104				
Number of unigenes in the combined data		32,575				
Total nucleotides of transcripts (bp)		122,947,955				
Total nucleotides of unigenes (bp)		34,155,642				
Mean length of transcripts (bp)		1,116				
Mean length of unigenes (bp)		1,048				
N50 of unigenes (bp)		1350				

Q30, base call accuracy of 99.9%; N50, the sequence length of the shortest unigene at 50% of the total genome length.

**Table 2:** Top 20 enriched Kyoto Encyclopedia of Genes and Genomes (KEGG) pathways of upregulated differentially expressed genes (DEGs).

Pathway	DEG Number	Pathway ID
Ribosome	58	ko03010
MAPK signaling pathway-plant	40	ko04016
Plant-pathogen interaction	40	ko04626
Plant hormone signal transduction	37	ko04075
Phenylpropanoid biosynthesis	28	ko00940
Starch and sucrose metabolism	25	ko00500
Flavonoid biosynthesis	23	ko00941
Fluid shear stress and atherosclerosis	23	ko05418
Phenylalanine metabolism	19	ko00360
Cancer-related pathways	18	ko05200
Protein processing in the endoplasmic reticulum	18	ko04141
Cellular senescence	16	ko04218
Endocytosis	16	ko04144
Glycolysis/Gluconeogenesis	16	ko00010
β-Alanine metabolism	15	ko00410
Calcium signaling pathway	14	ko04020
Oxytocin signaling pathway	14	ko04921
Phagosome	14	ko04145
Amino sugar and nucleotide sugar metabolism	13	ko00520
Arginine and proline metabolism	13	ko00330

**Table 3:** Top 20 enriched KEGG pathways of downregulated DEGs.

Pathway	DEG Number	Pathway ID
Folate biosynthesis	13	ko00790
Starch and sucrose metabolism	8	ko00500
Plant hormone signal transduction	7	ko04075
Brassinosteroid biosynthesis	6	ko00905
Phenylpropanoid biosynthesis	6	ko00940
Circadian rhythm - plant	5	ko04712
Cyanoamino acid metabolism	5	ko00460
Glyoxylate and dicarboxylate metabolism	5	ko00630
Protein processing in the endoplasmic reticulum	5	ko04141
Renin-angiotensin system	5	ko04614
β-Alanine metabolism	4	ko00410
Glycine, serine and threonine metabolism	4	ko00260
Lysosome	4	ko04142
Phenylalanine metabolism	4	ko00360
Photosynthesis	4	ko00195
Photosynthesis - antenna proteins	4	ko00196
Platinum drug resistance	4	ko01524
Protein digestion and absorption	4	ko04974
Purine metabolism	4	ko00230
Tropane, piperidine, and pyridine alkaloid biosynthesis	4	ko00960

**Table 4:** Expression profiles of anthocyanin biosynthetic genes.

Gene Name	Unigene ID	Gene Length	FPKM		Fold Change	log2Fold Change
			Wild Type	S7 Mutant		
*PAL1*	denphalae05809	2223	3.98	89.98	21.44	4.42
*PAL2*	denphalae05806	2093	12.98	196.34	14.13	3.82
*PAL3*	denphalae05808	2139	8.59	71.56	7.45	2.90
*C4H*	denphalae10925	1518	1.92	18.63	8.97	3.16
	denphalae10926 *	1518	6.09	36.67	5.50	2.46
*4CL*	denphalae18583 *	1731	15.16	79.17	4.78	2.26
	denphalae22607	1698	3.97	13.60	2.89	1.53
	denphalae27156	1473	0.63	3.23	5.15	2.36
	denphalae27157	1695	2.91	6.02	-	-
*CHS*	denphalae02657 *	1173	2.52	9.64	3.40	1.77
	denphalae02658	1170	10.50	36.49	3.15	1.65
	denphalae05188	1092	0.52	1.62	-	-
	denphalae11910	1188	102.38	659.26	5.62	2.49
*F3H*	denphalae02991	1137	98.69	245.15	2.07	1.05
*F3’H*	denphalae11915	1563	31.60	152.97	4.28	2.10
*DFR*	denphalae11241	1059	102.75	311.67	2.54	1.34
*ANS*	denphalae18276	1083	70.86	180.97	2.22	1.15

FPKM, fragments per kilobase of transcript per million mapped reads.

## Supplementary Materials

Supplementary materials can be found at https://www.mdpi.com/1422-0067/21/16/5653/s1. Figure S1. Images of *D. bigibbum.* Morphological phenotypes of typical wild type (WT) and the RB016-S7 mutant. Figure S2. Distribution of annotated sequences based on a Gene Ontology (GO) analysis. The GO functional classification assigned 17,498 unigenes to 41 subcategories under the three main GO categories of biological process, cellular component, and molecular function. The *x*-axis indicates the subcategories and the *y*-axis indicates the number of unigenes in each category. Figure S3. Numeric distribution of eggNOG annotations of unigenes. Letters on the *x*-axis refer to the categories on the right. The *y*-axis indicates the number of unigenes in the corresponding eggNOG category. Figure S4. Distribution of annotated sequences as assessed using a Kyoto Encyclopedia of Genes and Genomes (KEGG) pathway analysis. The *x*-axis indicates enriched KEGG pathways, and the *y*-axis represents the number of unigenes within each KEGG pathway. (A) Metabolism; (B) genetic information processing; (C) environmental information processing; (D) cellular processes; (E) organismal systems. Table S1. Significantly enriched GO terms. Table S2: Significantly enriched KEGG pathways. Table S3: Clusters of annotated GO terms in the biological process category enriched in up- and downregulated genes between WT and the RB016-S7 leaves. Table S4: Expression profiles of the regulatory genes for leaf color in *D. bigibbum*. Table S5: Gene-specific primers used for quantitative real-time PCR and *Agrobacterium* transient assay. Table S6: MYB2 gene sequence from *Dendrobium* orchids and *Cymbidium sinense* for *Agrobacterium* transient assay.

## Abbreviations

ANS	anthocyanidin synthase
4CL	4-coumarate CoA ligase
C4H	cinnamic acid 4-hydroxylase
CHI	chalcone isomerase
CHS	chalcone synthase
DFR	dihydroflavonol 4-reductase
F3H	flavanone 3-hydroxylase
F3′H	flavonoid 3′-monooxygenase
GO	gene ontology
KEGG	Kyoto Encyclopedia of Genes and Genomes
PAL	phenylalanine ammonia-lyase
WT	wild -type
