# Supplementary material for: Upregulation of the MYB2 Transcription Factor is Associated with Increased Accumulation of Anthocyanin in the Leaves of Dendrobium bigibbum
Source: Int J Mol Sci. 2020 Aug 6;21(16):5653. doi: 10.3390/ijms21165653 (PMC7460623; doi:10.3390/ijms21165653)
Supplement: Supplementary file 1 [file ijms-21-05653-s001.zip › TABLE S4.docx]

**Table S4. Expression profiles of transcript factor genes.**

|  | Gene name | Unigene ID | Gene | FPKR | | Fold | log2Fold |
| --- | --- | --- | --- | --- | --- | --- | --- |
|  |  |  | lengh | Wild-Type | S7 Mutant | Change | Change |
| MYB genes | MYB44 | denphalae02024 | 714 | 15.8 | 146.4 | 9.3 | 3.2 |
|  | MYB44 | denphalae08554 | 1,113 | 3.3 | 20.2 | 6.1 | 2.6 |
|  | MYB44 | denphalae08823 | 951 | 1122.9 | 3412.6 | 3.0 | 1.6 |
|  | MYB30 | denphalae13271 | 741 | 3.0 | 776.2 | 258.1 | 8.0 |
|  | MYB30 | denphalae13272 | 675 | 1.8 | 357.7 | 199.3 | 7.6 |
|  | MYB4 | denphalae13276 | 696 | 0.3 | 95.0 | 311.7 | 8.3 |
|  | MYB6-like | denphalae14728 | 501 | 0.6 | 11.8 | 19.5 | 4.3 |
|  | MYB75 | denphalae15711 | 1,842 | 1815.7 | 1512.7 | 0.8 | -0.3 |
|  | myb-related | denphalae18326 | 459 | 0.3 | 7.4 | 24.3 | 4.6 |
|  | myb-related | denphalae20511 | 891 | 18.3 | 735.5 | 40.1 | 5.3 |
|  | MYB2 | denphalae23717 | 513 | 349.3 | 969.8 | 2.8 | 1.5 |
|  | MYB2 | denphalae23718 | 513 | 352.0 | 954.6 | 2.7 | 1.4 |
|  | MYB2 | denphalae23719 | 873 | 309.8 | 1303.7 | 4.2 | 2.1 |
|  | myb-related | denphalae25812 | 531 | 74.1 | 17.6 | 0.2 | -2.1 |
|  | myb-related | denphalae25813 | 636 | 93.1 | 23.0 | 0.2 | -2.0 |
|  | MYB44 | denphalae31565 | 303 | 19.3 | 3.2 | 0.2 | -2.6 |
|  | MYB39 | denphalae32432 | 654 | 40.6 | 166.6 | 4.1 | 2.0 |
|  | MYB39 | denphalae32433 | 654 | 46.1 | 174.0 | 3.8 | 1.9 |
|  | MYB3 | denphalae32484 | 975 | 52.8 | 13.4 | 0.3 | -2.0 |
|  | MYB3 | denphalae32485 | 975 | 117.8 | 32.3 | 0.3 | -1.9 |
| bHLH genes | bHLH96 | denphalae02814 | 960 | 5.0 | 36.6 | 7.4 | 2.9 |
|  | bHLH1 | denphalae13088 | 1,362 | 82.8 | 377.9 | 4.6 | 2.2 |
|  | bHLH1 | denphalae13089 | 1,467 | 137.1 | 469.1 | 3.4 | 1.8 |
|  | bHLH1 | denphalae13090 | 1,575 | 138.4 | 532.2 | 3.8 | 1.9 |
|  | bHLH1 | denphalae13091 | 411 | 20.2 | 127.4 | 6.3 | 2.7 |
|  | bHLH1 | denphalae13092 | 573 | 15.3 | 73.7 | 4.8 | 2.3 |
|  | bHLH1 | denphalae13093 | 1,935 | 104.5 | 708.3 | 6.8 | 2.8 |
|  | bHLH1 | denphalae13094 | 1,425 | 131.9 | 429.7 | 3.3 | 1.7 |
|  | bHLH63 | denphalae15296 | 972 | 617.9 | 123.0 | 0.2 | -2.3 |
|  | bHLH153 | denphalae17497 | 693 | 96.3 | 326.0 | 3.4 | 1.8 |
|  | bHLH041 | denphalae18019 | 1,206 | 11.6 | 73.5 | 6.4 | 2.7 |
|  | bHLH-type-like | denphalae18144 | 1,503 | 51.0 | 536.3 | 10.5 | 3.4 |
|  | bHLH35 | denphalae21805 | 543 | 1.2 | 52.4 | 43.0 | 5.4 |
|  | bHLH113 | denphalae22329 | 354 | 43.9 | 142.3 | 3.2 | 1.7 |
|  | bHLH155 | denphalae25667 | 1,515 | 129.2 | 19.8 | 0.2 | -2.7 |
|  | bHLH92 | denphalae27054 | 666 | 24.6 | 160.7 | 6.5 | 2.7 |
|  | bHLH114 | denphalae28097 | 942 | 8.0 | 148.2 | 18.6 | 4.2 |
|  | bHLH114 | denphalae28098 | 741 | 3.0 | 79.2 | 26.4 | 4.7 |
|  | bHLH114 | denphalae28099 | 630 | 3.6 | 81.4 | 22.5 | 4.5 |
|  | bHLH62 | denphalae28316 | 1,365 | 35.2 | 158.4 | 4.5 | 2.2 |
|  | bHLH62 | denphalae28317 | 978 | 11.5 | 61.1 | 5.3 | 2.4 |
|  | bHLH148 | denphalae31473 | 843 | 2.6 | 114.7 | 43.5 | 5.4 |
|  | bHLH93 | denphalae31491 | 873 | 209.9 | 65.3 | 0.3 | -1.7 |
| WD40 | TTG1-like | denphalae04639 | 1,026 | 901.1 | 1064.8 | 1.2 | 0.2 |
| WRKY genes | WRKY | denphalae00817 | 1,638 | 212.9 | 937.4 | 4.4 | 2.1 |
|  | WRKY26 | denphalae00818 | 1,059 | 113.1 | 430.2 | 3.8 | 1.9 |
|  | WRKY | denphalae00819 | 1,692 | 192.5 | 1188.0 | 6.2 | 2.6 |
|  | WRKY24 | denphalae00820 | 345 | 8.2 | 101.7 | 12.4 | 3.6 |
|  | WRKY24 | denphalae00821 | 1,113 | 91.0 | 696.6 | 7.7 | 2.9 |
|  | WRKY53 | denphalae01062 | 879 | 0.9 | 85.3 | 93.9 | 6.6 |
|  | WRKY53 | denphalae01063 | 531 | 1.2 | 24.8 | 20.8 | 4.4 |
|  | WRKY49 | denphalae01146 | 345 | 159.8 | 35.2 | 0.2 | -2.2 |
|  | WRKY41-like | denphalae03544 | 582 | 6.5 | 170.2 | 26.1 | 4.7 |
|  | WRKY53 | denphalae03545 | 690 | 18.9 | 356.2 | 18.8 | 4.2 |
|  | WRKY41-like | denphalae03546 | 513 | 4.7 | 152.0 | 32.1 | 5.0 |
|  | WRKY53 isoform | denphalae03547 | 1,047 | 27.1 | 567.0 | 20.9 | 4.4 |
|  | WRKY70 isoform | denphalae03654 | 912 | 350.2 | 1551.6 | 4.4 | 2.1 |
|  | WRKY24 | denphalae06256 | 336 | 6.8 | 101.0 | 14.9 | 3.9 |
|  | WRKY24 | denphalae06259 | 360 | 5.3 | 138.1 | 25.8 | 4.7 |
|  | WRKY40 | denphalae06648 | 840 | 3.6 | 91.6 | 25.4 | 4.7 |
|  | WRKY24 | denphalae06841 | 402 | 104.6 | 397.9 | 3.8 | 1.9 |
|  | WRKY24 | denphalae06842 | 936 | 294.5 | 2054.5 | 7.0 | 2.8 |
|  | WRKY40 | denphalae12606 | 1,119 | 6.0 | 630.6 | 105.9 | 6.7 |
|  | WRKY40 | denphalae12607 | 618 | 0.6 | 104.3 | 171.0 | 7.4 |
|  | WRKY40 | denphalae12608 | 1,146 | 2.4 | 449.0 | 185.6 | 7.5 |
|  | WRKY51 isoform | denphalae12946 | 714 | 136.8 | 514.4 | 3.8 | 1.9 |
|  | WRKY51 isoform | denphalae12948 | 840 | 193.0 | 647.6 | 3.4 | 1.7 |
|  | WRKY50 | denphalae14038 | 639 | 436.5 | 129.4 | 0.3 | -1.8 |
|  | WRKY31 | denphalae16791 | 1,674 | 319.8 | 1221.5 | 3.8 | 1.9 |
|  | WRKY31 | denphalae16792 | 1,038 | 73.1 | 466.9 | 6.4 | 2.7 |
|  | WRKY41 | denphalae18208 | 948 | 277.4 | 1415.7 | 5.1 | 2.4 |
|  | WRKY40 isoform | denphalae25003 | 576 | 2.8 | 34.1 | 12.0 | 3.6 |
|  | WRKY40 isoform | denphalae25004 | 576 | 4.3 | 27.1 | 6.2 | 2.6 |
|  | WRKY40 isoform | denphalae25005 | 942 | 21.9 | 122.7 | 5.6 | 2.5 |
|  | WRKY70 | denphalae27315 | 495 | 7.6 | 76.7 | 10.1 | 3.3 |
|  | WRKY70 | denphalae27316 | 936 | 44.2 | 365.1 | 8.3 | 3.0 |
|  | WRKY48 | denphalae31867 | 963 | 132.5 | 1332.9 | 10.1 | 3.3 |
