# Supplementary material for: Upregulation of the MYB2 Transcription Factor is Associated with Increased Accumulation of Anthocyanin in the Leaves of Dendrobium bigibbum
Source: Int J Mol Sci. 2020 Aug 6;21(16):5653. doi: 10.3390/ijms21165653 (PMC7460623; doi:10.3390/ijms21165653)
Supplement: Supplementary file 1 [file ijms-21-05653-s001.zip › TABLE S6.docx]

**> Dendrobium hybrid cultivar cultivar Blue sapphine No. 3 MYB2 (KY039157.1)**

ATGGGAAAAAATCCGTGCTGCTTCAAGGAAGGGCTTAACAAGGGAGCATGGACTACTTCGGAAGACATGCTCTTGAAGGCTTTCGTCAATATTCATGGAGAAGGCAAATGGACGACGGTGCCACACAAAGCAGGGCTGAGAAGGTCGGGGAAGAGCTGTCGACTCCGATGGCTAAATTACCTGAGGCCCAACGTTAAACATGGAAACTTTTCCGAGGAAGAGGACGACCTCATCATCAGACTTCATAAACTCCTTGGCAATAGATGGTCTCTGATTGCTGGAAGGCTACCCGGTCGGACAGATAATGAAATAAAAAACTATTGGAACATAACCTTATGTAAGAAAGCAAGCTTTCAACATCAAATGCATCAGCCAAGCCGCCCAAGCATCATACAAAGACCTCCAACTTCTAATCTTGGATTTCCAACATCATCATCTACAACACAATCACAAGCTGCCACAAATGATACAACTTTGATCCTAACAACGGCCATAAGGTGCAATAAACTGGCTATTCCAATGCTACTTCCATCTTCTTCAACAAGTAAGCAGGAGATCCCAAACATGCAATTAGCAGAAGAGTCAATAGCTAAAGTGGCTAGTATGATTCCTGAAAGCAGCAAAGTTGGTCCCTTGGTAGAAGAAGATCTTTTTAAGGAACTATTTCAGGTGGAGGAGAATATGGTTTTGAATAACGACAAATTTAATGATGACAATATTGTTTCATTTCCTGATCAAGCAGCTGTAATGGAGTTTGAAAGATTACAAGATTTTGAGAAATGGATGCTGAATGATGAAGATGTTGATTGCCTTCCTCCTAATGATCAAATGCGTATGTTGACCTCTTTATTTGATATAGGAAGTGAATTCTAG

**> Dendrobium biggibum MYB2**

ATGGGAAGGAATTCATGTTGTTTCAAGGAAGGGCTTAACAAGGGAGCATGGAGTACTTCGGAAGACAAGCTCTTGAAGGCTTTCATCAATATTCATGGAGAAGGCAAATGGACGACGGTGCCATACAAAGCAGGGCTGAGAAGGTCTGGGAAGAGTTGCCGACTCCGATGGCTGAATTACCTGAGGCCGAACGTTAAACGTGGAAACTTTTTAGAGGAAGAGGACGACCTCATCATCAGGCTTCATAAACTCCTTGGCAATAGATGGTCTCTGATTGCTGGAAGGCTACCCGGTCGGACAGATAATGAAATAAAAAATTACTGGAACACAACCTTATATAAGAAAGCAATATTTCAACATCAAATGCGTCAGCCAAGCCGCCCAAGCATCATACAAAGACCTCCAACTTCTAATCTTGTATTTCCAACATCATCATCTACAACACAATCACAAGCTGCTATAAATGATACAACTTTGATCCTAACAACGGCCATAAGGTGCAATAAAGTGGCTATTCCAATGCTACTTCCATCTTCTTCAACAAGTAAGCAGGAGATCCCAAACATGCAATTAGCAGAAGAGTCAATAGCTGAAGTGGCTAGTGAGATGCCTGAAAGCAGCAAAGTAGGTCCCATGGTAGAAGAAGATCTTCTTAAGGAACTGTTTCAGGTGGAAGAGAATATGGTTTTGAACAACGACAAATTTAATGATGACAATATTGTTGCATTTCCTGATCAGGCGTCTGTAATGGAGTTTGAAAGATTACAAGATTTTGAGAAGTGGATGCTGAATGATGAAGATGTTGATTGCCTTCCTCCTGATGATCAAATGCATATGTTGACCTCTTTATTTGATATAGGAAGTGAATTCTAG

**>Dendrobium catenatum MYB2 (XM_020817158.2)**

ATGGGAAGGAATTCGTGCAGTTTCAAGGAAGGGCTTAACAAAGGAGCATGGACTACTGTGGAAGACAAGCTCTTGACGGCTTTCATCAATATTCATGGAGAAGGCAAATGGACGACTGTGCCCTACAAAGCAGGATTGAAAAGATCTGGGAAGAGCTGTCGGCTCCGATGGCTAAATTATCTAAGGCCCAACGTTAAACGTGGAAACTTTTCCGAGGAAGAGAACGACCTCATCATCAGGCTTCATAAGCTCCTTGGCAATAGATGGTCATTGATCGCTGGAAGACTACCAGGTCGAACAGATAATGAAATAAAAAATTATTGGAATACAACCTTAGGTAAGATTGCAAGCTTTCAACATCAACCACATCAGCCAAGCCGCCCAAGCATCATACAAAGGCCTCCTGCTTCTAATCTCATATTTCCATCACCATCATCTTCAACACCATCACAAGCTACAAATAATGATAAAACTTTAATCCGAACAACAGCCATAAGGTGCAATAATGTGATTATTCCAATGCAGCTACCATCTTCTTCAACAAGCAAGAAGGATATCCCAAGCATGCAATTAGCAGAAGAGTCAATGGCTAAGGTGGCTAGTGACATGCCCAAAAGTAGCAAAGTTGGTCCCATGGTCGAAGAAGAGCTTTTTAAGGAATTTTTTCAGTTGGATGAGAATATGGTTTTGAACTACAACAGCTTCGATGATGACACCAATGTTGCATTTCCTACACAGGCGAGTGTAATGGAGTTTTAA

**>Dendrobidum hybrid**

ATGGGAAGGAATTCGTGCAGTTTCAAGGAAGGGCTTAACAAAGGAGCATGGACTACTGTGGAAGACAAGCTCTTGACGGC

TTTCATCAATATTCATGGAGAAGGCAAATGGACGACTGTGCCCTACAAAGCAGGGTTGAAAAGATCTGGGAAGAGCTGTCGGCTCCGATGGCTGAATTACTTAAGGCCCAACGTTAAACGTGGAAACTTTTGCGAGGAAGAGGACGACCTCATCATCAGGCTTCATAAGCTCCTTGGCAATAGATGGTCATTGATTGCTGGAAGAATACCAGGTCGAACAGATAATGAAATAAAAAATTATTGGAATACAACCTTAGGCAAGATTGCAAGCTTTCAACATCAAAGACATCAGCCATGCCGCCCAAGCATCATGCAAAGGCCTCCTGCTTATAATCTCATAGCTCCATCACCATCATGTTCAACACCATTGCAAGCTACAAAAAATGATAATGCTTTAATCCGAACAACGGCCATAAGGTGCAATAATGTAGCTATTCCAAAGCAGCTTCCATCTTCTTCAACAAGCAACCCGGATATCCCAAGCATGCAATTAGCAGAAG

ATTCAATTGTTAAGGTGGCTAGTGAGATGCCCGAAAGTAGCAAAGTTGGTCCCATGGTAGAAGAAGAGCTATTTATGGAATTGTTTCAGTTGGAAGAGAATATTGTTTTGAACTACAACACCTTCGATGATGACAACAATGATGCATTTCCTGCTCAGGCGAGTGTAATGGAGTTTTAA

**>Dendrobium candidum MYB2**

ATGGGAAGGAATTCGTGCAGTTTCAAGGAAGGCCTTAACAAGGGAGCATGGACTACTGCGGAAGACAAGCTCTTGACGGC

TTTCATCAATATTCATGGAGAAGGCAAATGGACGACTGTGCCGTACAAAGCAGGGTTGAAAAGATCTGGGAAGAGCTGTCGGCTTCGATGGCTAAATTACCTAAGGCCCAACGTTGAACGTGGAAACTTTTCCGAGGAAGAGGACGACCTCATCATCAGGCTTCATAAGCTCCTTGGCAATAGATGGTCATTGATTGCTGGAAGAATACCAGGCCGAACAGATAATGAAATAAAAAATTATTGGAATACAACCTTAGGCAAGATTGCAAGCTTTCAACATCAAAGACATCAGCCATGCCGCCCAAGCATCATGCAAAGGCCTCCTGCTAATAATCTCATAGTTCCATCACCATCATGTTCAACACCATTGCAAGCTACAAAAAATGATAATACTTTAATCCGAACAACGGCAATAAGGTGCAATAATGTAGCTATTCCAAAGCAGCTTCCATCTTCTTCAACAAGCAATCCGGATATCCCAAGCATGCAATTAGCAGAAGATTCAATGGTTAAGGTGGCTAGTGAGATGCCCGAAAGTAGCAAAGTTGGTCCCATGGTAGAAGAAGAGCTATTTAAGGAATTGTTTCAGTTGGAAGAGAATATTGTTTTGAACTACAACACCTTCGATGATGACAACAATGATGCATTTCCTGCTCAGGCGAGTGTAATGGAGTTTTAA

**>Dendrobium nobile MYB2**

ATGGGGAGGCACTCTTGTTGCTACAAGCAGAAGTTGAGGAAGGGCTTGTGGTCTCCTGAGGAGGATGAGAAGCTCCTCAAGCATATCACAAAGTATGGCCATGGATGCTGGAGCTCAGTGCCTAAGCTTGCAGGCCTGCAGAGATGCGGGAAAAGCTGTAGGCTGCGGTGGATTAACTATCTGAGGCCTGATTTGAAGAGAGGAACTTTCTCGCAGCAGGAGGAGAACCTCATCATTGAGCTTCACGGAATTTTAGGAAACAGGTGGTCTCAGATTGCAGCACAATTGCCCGGAAGAACTGATAACGAGATAAAGAACTTGTGGAATTCTTGCATCAAGAAGAAGCTTAGGCAGAGAGGCATAGACCCCAACACGCACAAGCCGTTCGCTGAAATCGAAGGACAGCTCGCCGAAATCGACTCCGCCGATCAACCAGCTGCAGAACCAGAACCGATGAAGCATCCTGTGCAGCCACCGGAGACTGGTATTCCAAATAATTCTGTAAGGCCAGCCAATTCTTCAGCAGCTGGCTACTTCTCTCTTCCAAATTTAACTTACAGCCCTGATTGTGGAAGTAACATTGGCCAAATCCAGCAGTTTTGGTATAATCAAAGCGGAAAAATTTTTAGCAACAACAACTCCAACTCTGAGTTCAGCTTTAATTCCGCATCTAGCTTACTCCCGTCTGTTCCCAGATCCACTGTTTCAACTTCGAAGGAGCCGAAGCCTCTAACTTCTCTCCTAATGGAGAATTCTCCATCAGGATTCTACTGGGAGACAGGGAATTCAAGCAACAGCAGCGCCAGCAGTGGGAGCAATGATCCCCTGTTCGACGGAAGCATCTTTCAATGGACCCAGCTGATTCCAGAGAGAGATACAAATGTCCAGCTCCAGGGAGAAACTGATGACCTCAAATGGTCAGAATATCTTAATGGCTCAGTTCCATTA

**>Cymbidium. sinense MYB2**

ATGGGGAGGCACTCTTGCTGCTACAAGCAGAAGTTGAGGAAGGGATTATGGTCTCCTGAGGAGGACGAGAAGCTTCTCGAGCATATCACAAAGTATGGTCATGGATGCTGGAGCTCAGTGCCTAAGCTTGCAGGCCTGCAGAGATGTGGTAAAAGCTGCAGGCTGAGGTGGATAAACTATCTCAGGCCTGATTTAAAGCGAGGAACTTTCTCACAGGAGGAGGAGAACCTCATCATTGACCTTCACGGAGTGTTAGGAAACAGGTGGTCTCAGATTGCAGCACAACTGCCAGGAAGAACCGATAATGAGATAAAGAACTTGTGGAACTCTTGCATTAAGAAGAAGTTGAGGCAGAGAGGGATAGACCCCAACACACACAAGCCGCTCGCTGAAATCGACGAACAGCGGGAGAAAATAGCCGAAATTGACTCCTCCGGCCACCCAACTCCAGAACCAGCAAAGCAGACCGAACAGCCCGCAGAGACTGCTGTTCTGAACAATTTTGTGAGGCCAGCCAATTCTTCAGCAGCTAGTTACTACTCTCTTCCAAATTTAACTTACAGCGATGAGTGCGGAAATAATATTGGCCTAATCCAGCAGTTTTGGTTCAATCAAAGCAGCAAATTTTTCAACGCCATCAACCCAAACTCTGAGTTCAGTTTTAATTCAGTTTCTAGCTTACTGCCATCGGTTCCGCGGTCTACCCTTTCAACTTCTAAGGAGCTGAAGCCTCTAACCAATCTCCTGATGGAGAATTCTCCATCAGGATTCTACTGGGAGGCAGGGAATTCAAGCAACAGCAGCGCGAGCAGTGGGAGCAATGATCCCCTGTTTGATGGAAGCATCTTTCCATGGACTCAGCTGATGCCTGAAAGAGATACAAATATCCAGCTCCATGGAGAGACTGAGGACCTCAAATGGTCAGAATATCTTAATGGCTCCATTCCATTA
