# Supplementary figures and images for: Upregulation of the MYB2 Transcription Factor is Associated with Increased Accumulation of Anthocyanin in the Leaves of Dendrobium bigibbum
Source: Int J Mol Sci. 2020 Aug 6;21(16):5653. doi: 10.3390/ijms21165653 (PMC7460623; doi:10.3390/ijms21165653)

**Figure S1**

**RB016A-WT**

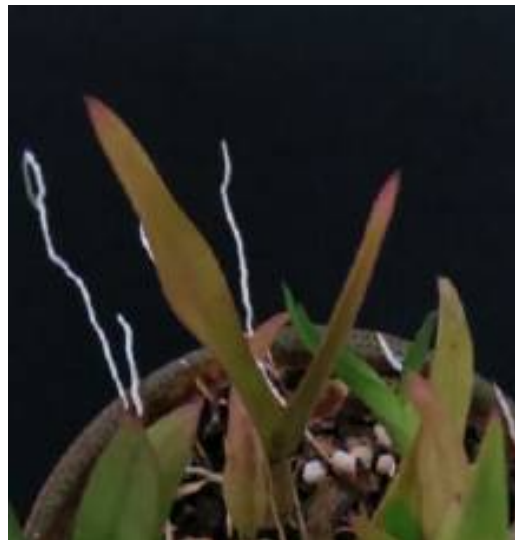

**RB016A-S7**

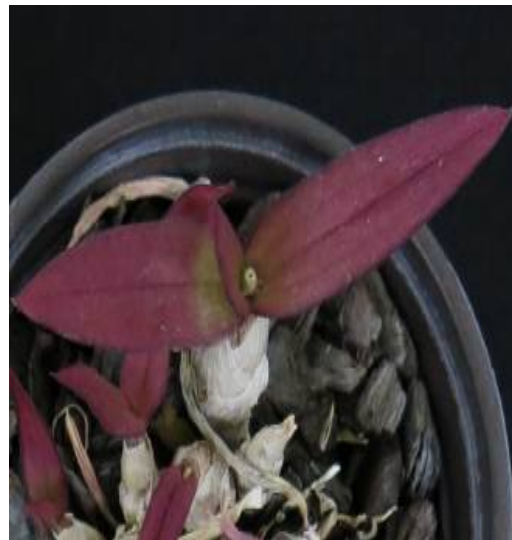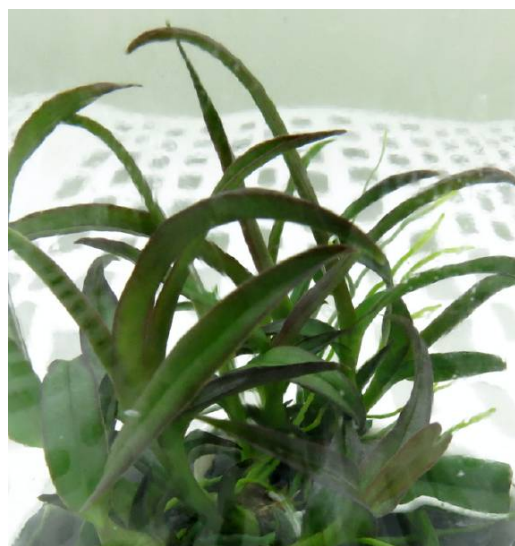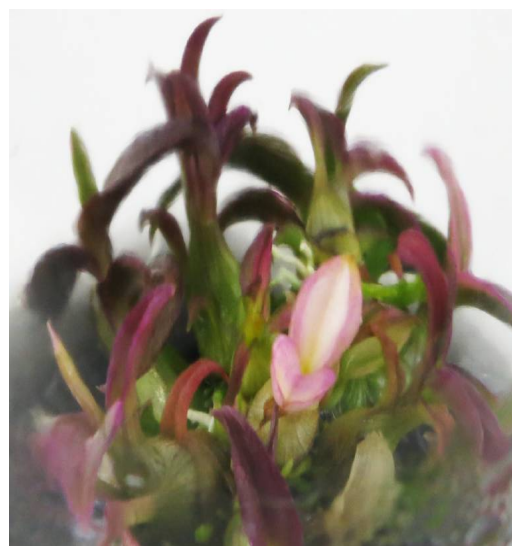

GO classification

Figure S2

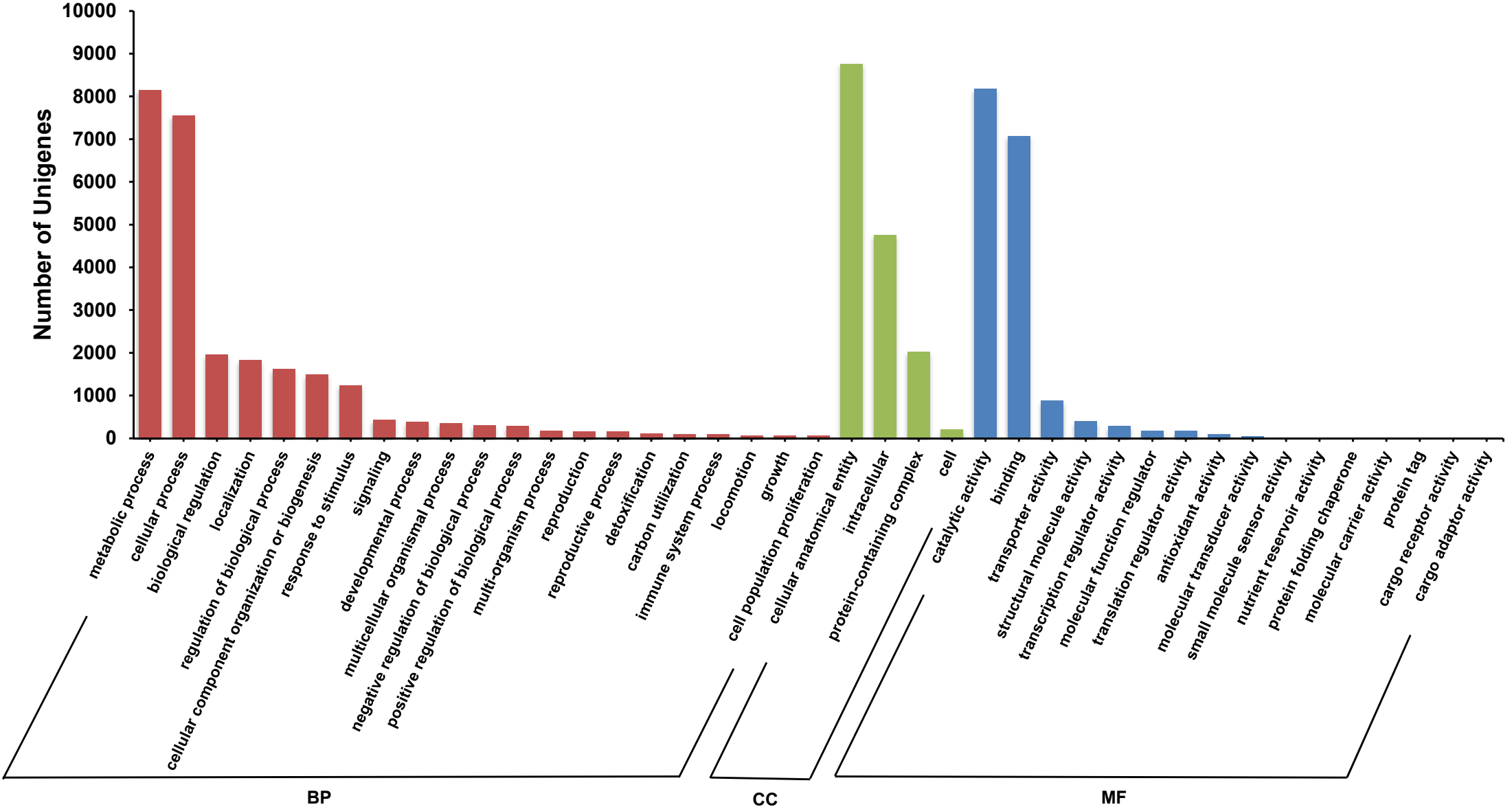

**Figure S3**

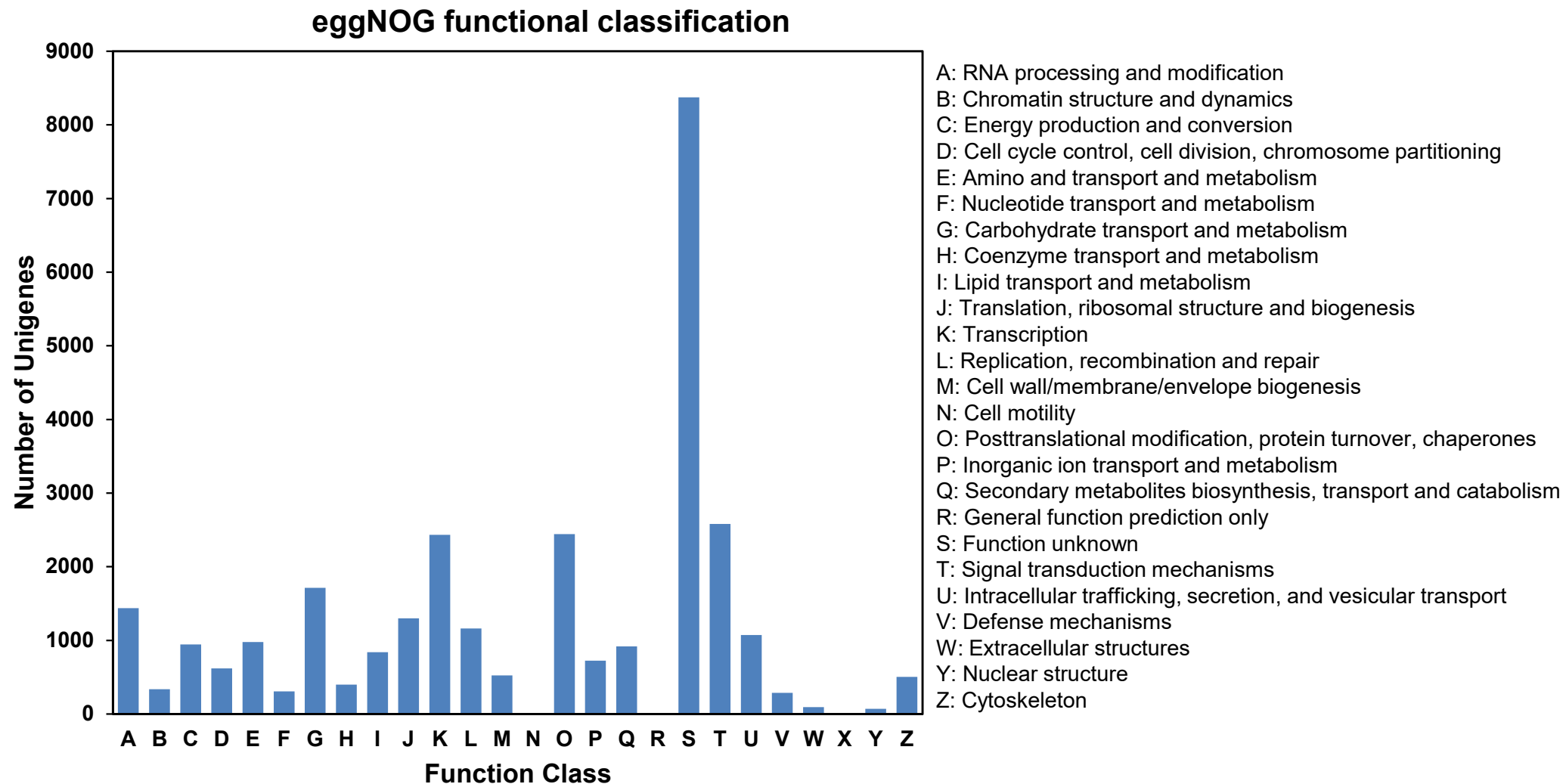

Figure S4

## KEGG Classification

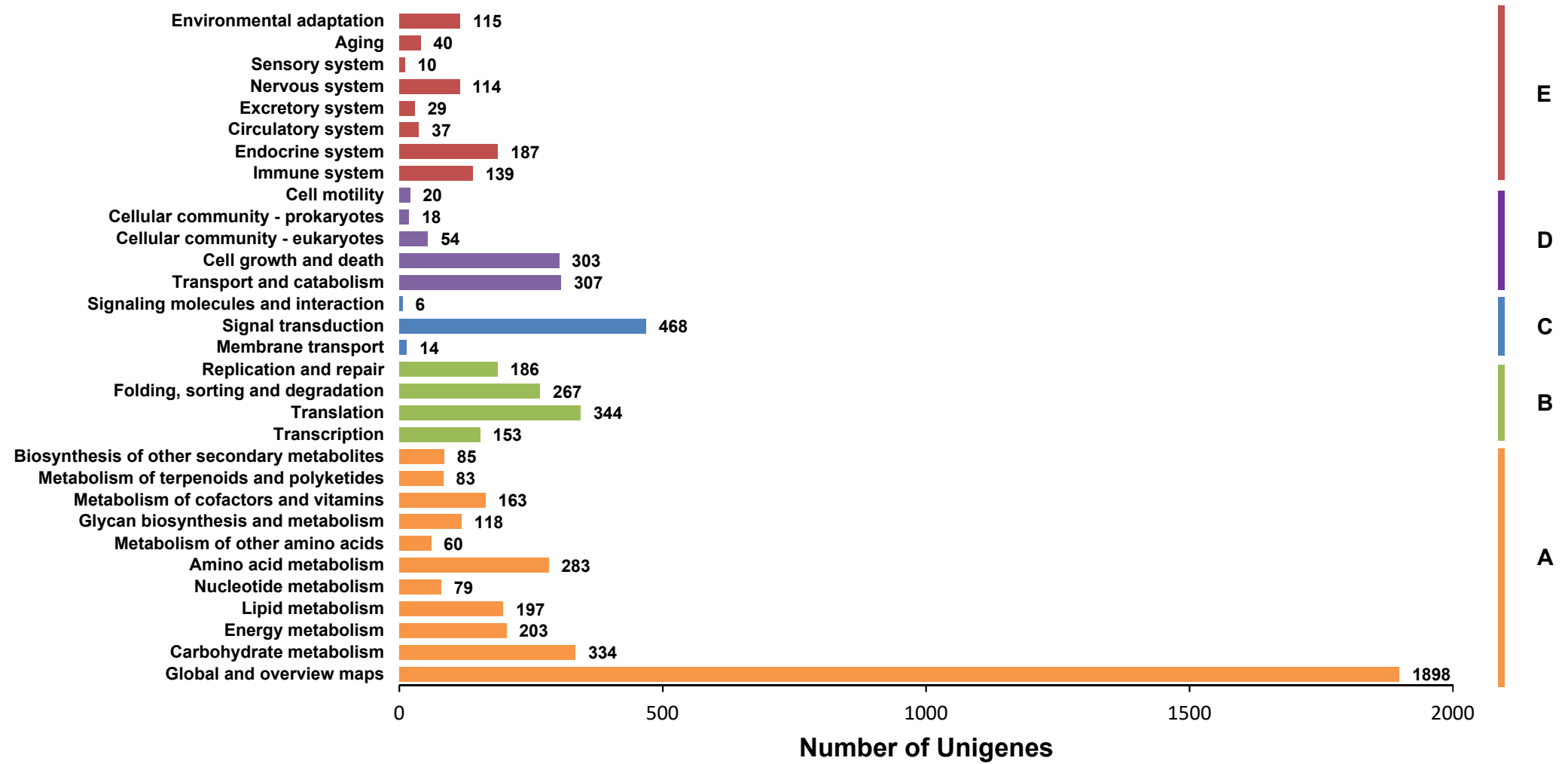

Supplement: Supplementary file 1 [file ijms-21-05653-s001.zip › Figure S.pdf]
